# Na-ion Storage Performances of FeSe_x_ and Fe_2_O_3_ Hollow Nanoparticles-Decorated Reduced Graphene Oxide Balls prepared by Nanoscale Kirkendall Diffusion Process

**DOI:** 10.1038/srep22432

**Published:** 2016-02-29

**Authors:** Gi Dae Park, Jung Sang Cho, Jung-Kul Lee, Yun Chan Kang

**Affiliations:** 1 Department of Materials Science and Engineering, Korea University, Anam-Dong, Seongbuk-Gu, Seoul 136-713, Republic of Korea; 2 Department of Chemical Engineering, Konkuk University, 1 Hwayang-Dong, Gwangjin-Gu, Seoul 143-701, Republic of Korea

## Abstract

Uniquely structured FeSe_x_-reduced graphene oxide (rGO) composite powders, in which hollow FeSe_x_ nanoparticles are uniformly distributed throughout the rGO matrix, were prepared by spray pyrolysis applying the nanoscale Kirkendall diffusion process. Iron oxide-rGO composite powders were transformed into FeSe_x_-rGO composite powders by a two-step post-treatment process. Metallic Fe nanocrystals formed during the first-step post-treatment process were transformed into hollow FeSe_x_ nanoparticles during the selenization process. The FeSe_x_-rGO composite powders had mixed crystal structures of FeSe and FeSe_2_ phases. A rGO content of 33% was estimated from the TG analysis of the FeSe_x_-rGO composite powders. The FeSe_x_-rGO composite powders had superior sodium-ion storage properties compared to those of the Fe_2_O_3_-rGO composite powders with similar morphological characteristics. The discharge capacities of the FeSe_x_- and Fe_2_O_3_-rGO composite powders for the 200^th^ cycle at a constant current density of 0.3 A g^−1^ were 434 and 174 mA h g^−1^, respectively. The FeSe_x_-rGO composite powders had a high discharge capacity of 311 mA h g^−1^ for the 1000^th^ cycle at a high current density of 1 A g^−1^.

Transition metal chalcogenides (metal sulfides, selenides, and tellurides) have attracted considerable attention owing to their excellent properties and wide applications in various application fields including energy storage^1^,^2^,^3^,^4^,^5^,^6^,^7^,^8^,^9^,^10^,^11^,^12^,^13^,^14^,^15^,^16^,^17^,^18^,^19^,^20^,^21^,^22^,^23^. Transition metal sulfides with various compositions have been studied as efficient anode and cathode materials for lithium- and sodium-ion batteries^10^,^11^,^12^,^13^,^14^. Recently, metal selenides have also been studied as anode materials for lithium- and sodium-ion batteries^15^,^16^,^17^,^18^,^19^,^20^,^21^,^22^,^23^. Metal selenides showed better electrochemical properties than their corresponding metal oxides for anode materials for Na-ion batteries (NIBs).

Nanostructured materials of metal selenides, such as MoSe_x_, NiSe_x_, FeSe_x_, SnSe_x_, CuSe_x_, and GeSe_x_, prepared mainly by a liquid solution process, have been studied to improve their sodium-ion storage properties by improving the structural stability during repeated sodium charge and discharge processes^19^,^20^,^21^,^22^,^23^. The combination of nanostructured metal selenides and carbon-based materials, such as amorphous carbon, graphitic carbon, and graphene, is considered an effective method for developing efficient NIB anode materials^22^,^23^. In particular, graphene and reduced graphene oxide (rGO), which have superior electrical conductivity and high mechanical strength compared to other carbon-based materials, have been successfully applied as support materials of nanostructured metal selenides, including nanopowders, nanoflowers, and nanoplates^24^,^25^,^26^,^27^.

Among the metal selenides, iron selenides, which are known to exist, i.e., FeSe_2_ (cubic and orthorhombic structure), FeSe (hexagonal and tetragonal), and Fe_7_Se_8_ (trigonal), have relatively high conductivity and are more advantageous as electrode materials for NIBs because iron is abundant, inexpensive, and environmentally friendly. Zhang *et al*. reported FeSe_2_ microspheres assembled by nanooctahedra prepared via a simple hydrothermal method. The FeSe_2_ microspheres delivered a stable discharge capacity of 372 mA h g^−1^ after 2000 cycles at 1 A g^−1 ^28. However, to the best of our knowledge, the synthesis of nanostructured iron selenide materials and their electrochemical properties as NIB anode materials have not been reported. In addition, iron selenide-graphene (or rGO) composite materials have also not been studied.

In this study, uniquely structured FeSe_x_-rGO composite powders were prepared by spray pyrolysis and subsequent selenization. Iron oxide-rGO composite powders prepared by spray pyrolysis were transformed into FeSe_x_-rGO composite powders, in which FeSe_x_ hollow nanoparticles were uniformly distributed throughout the rGO matrix. The iron oxide transformed into hollow FeSe_x_ nanoparticles through metallic iron nanopowders by a two-step post-treatment. Densely structured metallic iron nanopowders were transformed into hollow FeSe_x_ nanoparticles by the well-known nanoscale Kirkendall diffusion process during the selenization process by applying H_2_Se gas. The electrochemical properties and Na-ion storage of the uniquely structured FeSe_x_-rGO composite powders were compared with Fe_2_O_3_-rGO composite powders with similar morphological characteristics.

## Results and Discussion

**Figure 1:**
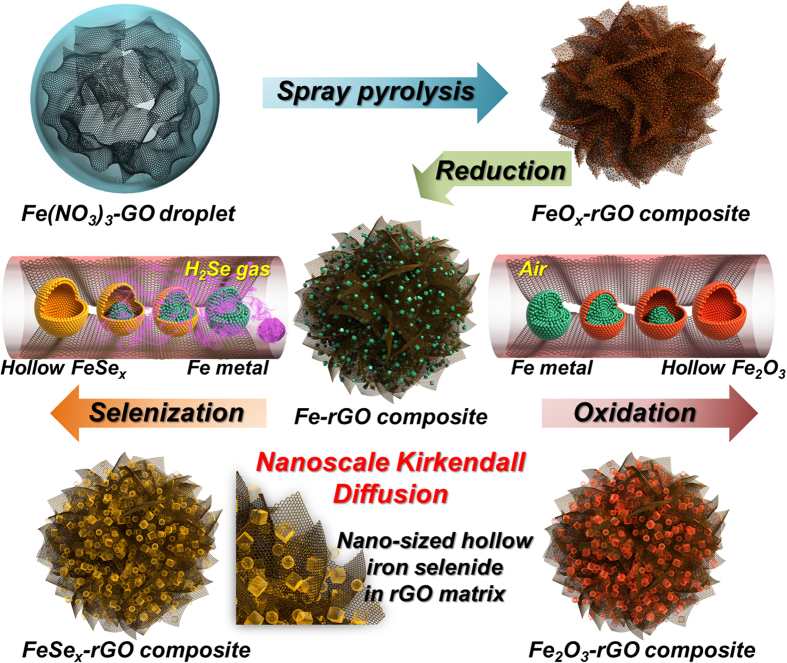
Formation mechanism of the hollow FeSe_x_ nanoparticles- and the hollow Fe_2_O_3_ nanoparticles-decorated rGO composite powders by nanoscale Kirkendall diffusion.

The formation mechanism of the hollow FeSe_x_ nanoparticles-decorated rGO composite and the hollow Fe_2_O_3_ nanoparticles-decorated rGO composite powders by spray pyrolysis with nanoscale Kirkendall diffusion is described in Fig. 1. Crumpled rGO powders decorated with iron oxide nanocrystals were prepared by the spray pyrolysis from a colloidal solution containing GO nanosheets and iron nitrate nonahydrate. Iron oxide-rGO composite powders were transformed into FeSe_x_-rGO composite powders, in which hollow FeSe_x_ nanoparticles were uniformly distributed throughout the rGO matrix, by a two-step post-treatment process. The first step of the post-treatment process at 400 ^o^C under a reducing atmosphere produced metallic iron-rGO composite powders, in which metallic Fe nanocrystals were uniformly distributed throughout the rGO matrix. The second step of the post-treatment process at 300 ^o^C under H_2_Se gas produced uniquely structured FeSe_x_-rGO composite powders. Metallic Fe nanocrystals were transformed into hollow FeSe_x_ nanoparticles during the selenization process by the well-known nanoscale Kirkendall diffusion process. In the early stage of the selenization process, a gas impermeable FeSe_x_ thin layer was formed over the metallic Fe nanocrystals. Subsequently, ion diffusion became the dominant process for further selenization. Fe cations with small radii (Fe^2+^ = 76 pm, Fe^3+^ = 65 pm) diffused outward more quickly than the larger selenium anions (184 pm) diffused inward. A new layer of iron selenide formed over the surface of the preformed FeSe_x_ layer. The diffusion out of the Fe cations during the selenization process formed the nanovoids inside the nanoparticle. The nanovoids formed inside the nanoparticle by nanoscale Kirkendall diffusion segregated into one nanovoid by complete transformation of metallic Fe into FeSe_x_ by selenization process. Finally, FeSe_x_-rGO composite powders, in which hollow FeSe_x_ nanoparticles were uniformly distributed throughout the rGO matrix, were prepared by a simple two-step post-treatment process at low temperatures. In the same way, the hollow Fe_2_O_3_ nanoparticles were prepared by nanoscale Kirkendall diffusion segregated into one nanovoid by complete transformation of metallic Fe into Fe_2_O_3_ by oxidation process at air atmospheres.

**Figure 2:**
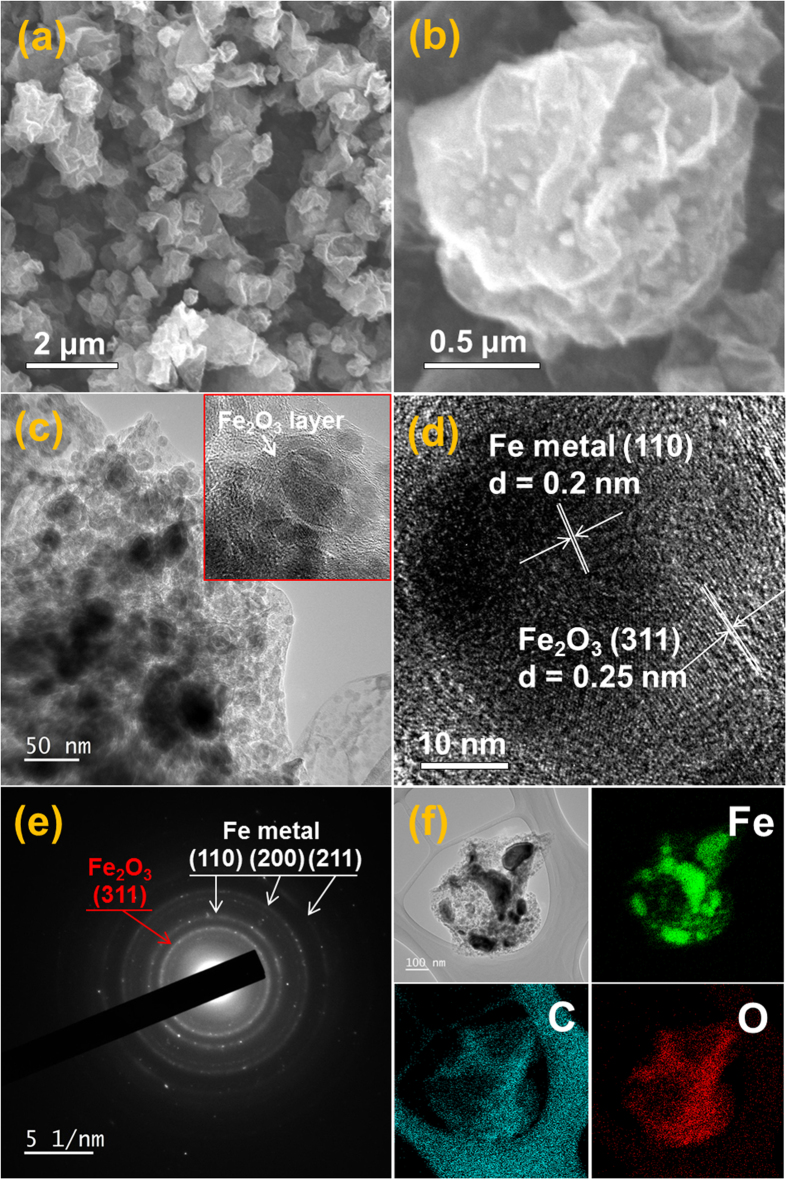
Morphologies, SAED pattern, and elemental mapping images of metallic iron-decorated rGO composite powders: (**a**,**b**) SEM image, (**c**) TEM image, (**d**) HR-TEM image, (**e**) SAED pattern, and (**f**) elemental mapping images.

The morphologies of the powders obtained before and after reduction are shown in Figs S2 and 2, respectively. The powders directly prepared by spray pyrolysis had non-crystalline structure, as shown by the XRD pattern in Fig. S3, because of the short residence time of the powders inside the hot wall reactor maintained at 400 °C for 14 s. The sharp peaks of the metallic Fe phase were observed in the XRD pattern of the powders obtained after reduction. The crumpled structure formed by spray pyrolysis was still maintained even after the reduction process, as shown by SEM images in Figs S2 and 2. The iron oxide nanocrystals covered with rGO sheets were not observed in the high-resolution SEM image shown in Fig. S2b. However, the ultrafine nanocrystals distributed inside the crumpled rGO were observed in the high-resolution SEM image shown in Fig. 2b. The TEM images shown in Fig. 2 revealed the metallic iron-rGO composite powders. Ultrafine nanopowders with a core-shell structure were uniformly distributed throughout the crumpled rGO matrix. The ultrafine metallic Fe nanocrystals had high oxidation characteristics even at a room temperature in air. Therefore, the surface oxidation of the metallic Fe nanocrystals dispersed within the rGO matrix formed the Fe_2_O_3_ layer. The further oxidation by nanoscale Kirkendall diffusion formed the void ring within the nanopowders, as indicated by arrows in Fig. 2c. The high resolution TEM image in Fig. 2d showed clear lattice fringes separated by 0.20 and 0.25 nm, which correspond to the (110) and (311) crystal planes of metallic Fe and Fe_2_O_3_ phases, respectively. The SAED pattern and elemental mapping images shown in Fig. 2e,f, respectively, also confirmed the formation of Fe_2_O_3_-coated metallic Fe nanocrystals by oxidation of metallic Fe nanocrystals.

**Figure 3:**
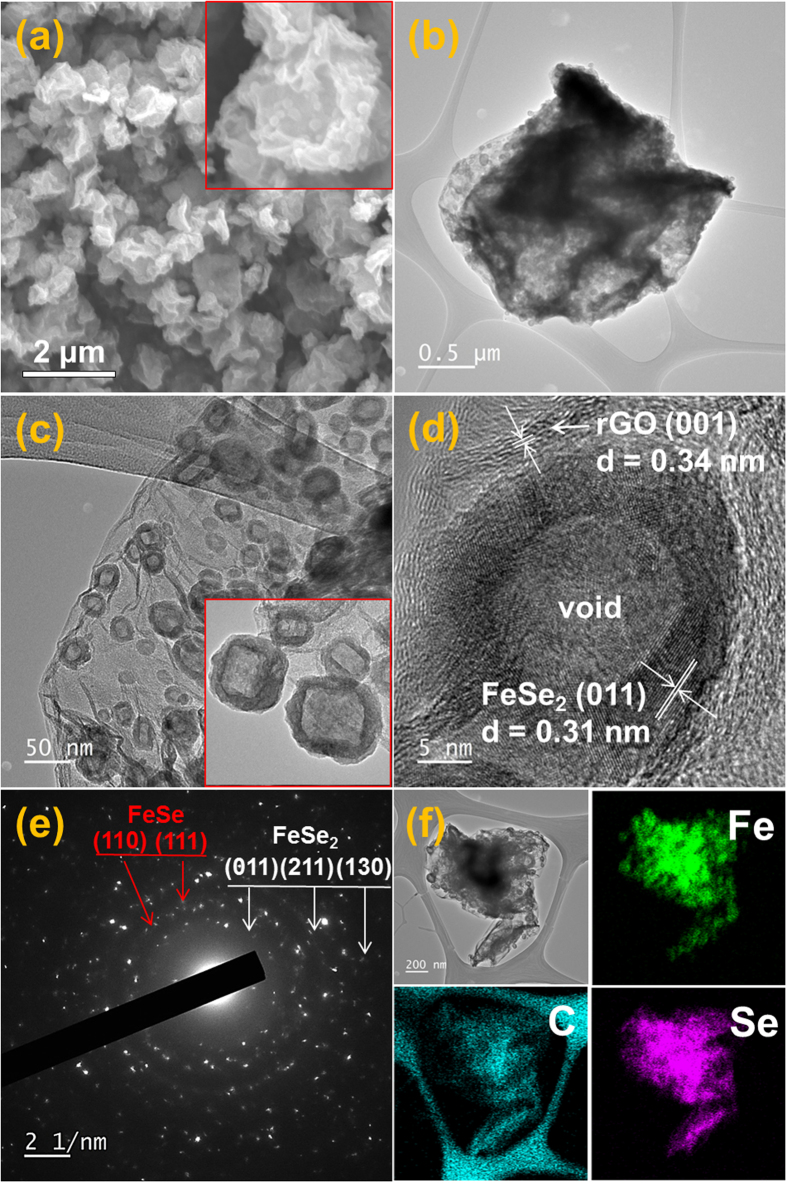
Morphologies, SAED pattern, and elemental mapping images of the hollow FeSe_x_-decorated rGO composite powders obtained by nanoscale Kirkendall diffusion: (**a**) SEM image, (**b**,**c**) TEM images, (**d**) HR-TEM image, (**e**) SAED pattern, and (**f**) elemental mapping images.

The morphologies and elemental mapping images of the FeSe_x_-rGO composite powders obtained after selenization at 300 °C for 6 h with the metallic iron-rGO composite powders are shown in Fig. 3. The SEM image shown in Fig. 3a shows morphologies similar to those of the metallic Fe-rGO composite powders. However, the TEM images shown in Fig. 3b–d show the unique structure of the FeSe_x_-rGO composite powders, in which hollow nanoparticles formed by nanoscale Kirkendall diffusion are uniformly distributed throughout the rGO matrix. The high-resolution TEM image in Fig. 3d shows clear lattice fringes separated by 0.31 and 0.34 nm, which correspond to the (011) and (001) crystal planes of the FeSe_2_ phase and rGO, respectively. Some of the hollow FeSe_x_ nanoparticles had a rectangular shape in the TEM image, shown as an inset image in Fig. 3c. The metallic Fe crystal growth during the long reduction process changed the spherical morphology into a rectangular parallelepiped morphology. The XRD pattern shown in Fig. S3 revealed the complete conversion of the metallic Fe into iron selenide with mixed crystal structures of FeSe and FeSe_2_ phases after selenization for 6 h. The SAED pattern and elemental mapping images shown in Fig. 3e,f, respectively, also confirmed the formation of FeSe_x_-rGO composite powders by complete selenization.

**Figure 4:**
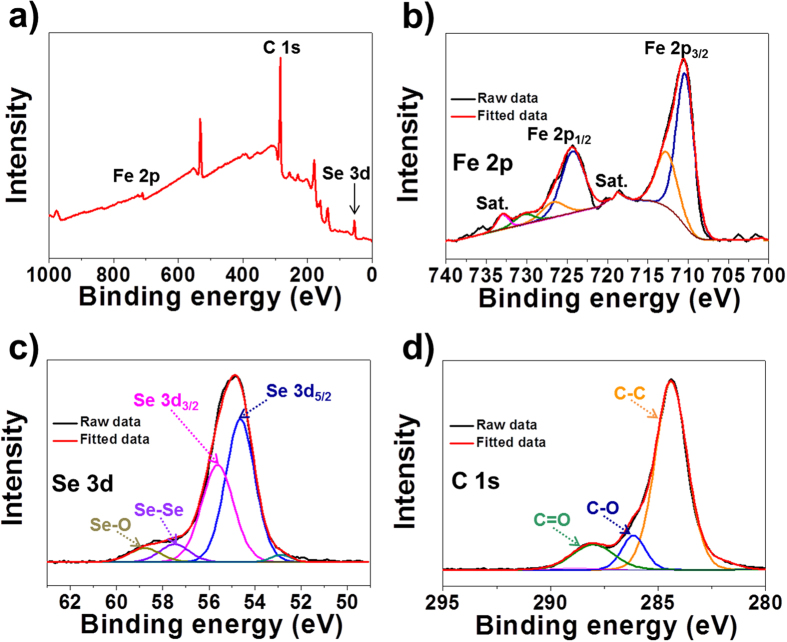
XPS spectra of the hollow FeSe_x_-decorated rGO composite powders: (**a**) wide-scan XPS spectrum, (**b**) Fe 2p, (**c**) Se 3d, and (**d**) C 1 s.

The chemical state and molecular environment of the XPS spectra of the FeSe_x_-rGO composite powders obtained after selenization at 300 °C for 6 h were characterized by X-ray photoelectron spectroscopy (XPS). The XPS survey spectrum in Fig. 4a of the FeSe_x_-rGO composite powders confirmed the presence of Fe, Se, and C signals. In the Fe 2p spectrum of the FeSe_x_-rGO composite powders, shown in Fig. 4b, the main peaks observed occurred at binding energies of 710.5 eV for Fe 2p_3/2_ and 724.2 eV for Fe 2p_1/2_; these are characteristic of iron selenide (FeSe) and two shake-up satellites^30^. The Se 3d spectrum of the FeSe_x_-rGO composite powders, shown in Fig. 4c, showed main two peaks located at 54.55 eV (Se 3d_5/2_) and 55.31 eV (Se 3d_3/2_), which are also consistent with iron selenides (FeSe and FeSe_2_)^31^. The Se–Se and Se-O bonds observed in Fig. 4c revealed the existence of metallic Se and SeO_2_ impurities formed during the selenization process^31^. The C1s peaks shown in Fig. 4d can be attributed to sp^2^-bonded carbon (C–C), epoxy and alkoxy groups (C–O), and carbonyl and carboxylic (C=O) groups, which correspond to peaks at 284.9, 286.7, and 287.6 eV, respectively^32^. The sharp XPS peak at 284.6 eV indicated the thermal reduction of GO into rGO during the preparation process of the FeSe_x_-rGO composite powders. The TG curve of the FeSe_x_-rGO composite powders shown in Fig. S4 revealed a one-step weight increase and one-step weight loss for temperatures below 800 °C. The conversion reaction of FeSe_x_ into FeSeO_x_ resulted in a weight increase at 150 °C. The weight loss observed at around 350 °C was attributed to the decomposition of FeSe_x_ and FeSeO_x_ into Fe_2_O_3_ and the combustion of rGO. A rGO content of 33% was estimated from the TG analysis of the FeSe_x_-rGO composite powders. The morphologies of the FeSe_x_-rGO composite powders obtained after selenization at 300 °C for 1 h are shown in Fig. S5. The conversion of the metallic Fe nanocrystals into FeSe_x_ hollow nanopowders by nanoscale Kirkendall diffusion occurred even at a short selenization time of 1 h. The overall FeSe_x_ nanopowders decorated within the rGO matrix had spherical and hollow morphologies. The XRD pattern of the composite powders obtained after selenization for 1 h shown in Fig. S6 revealed the minor peaks of the Fe_2_O_3_ phase.

**Figure 5:**
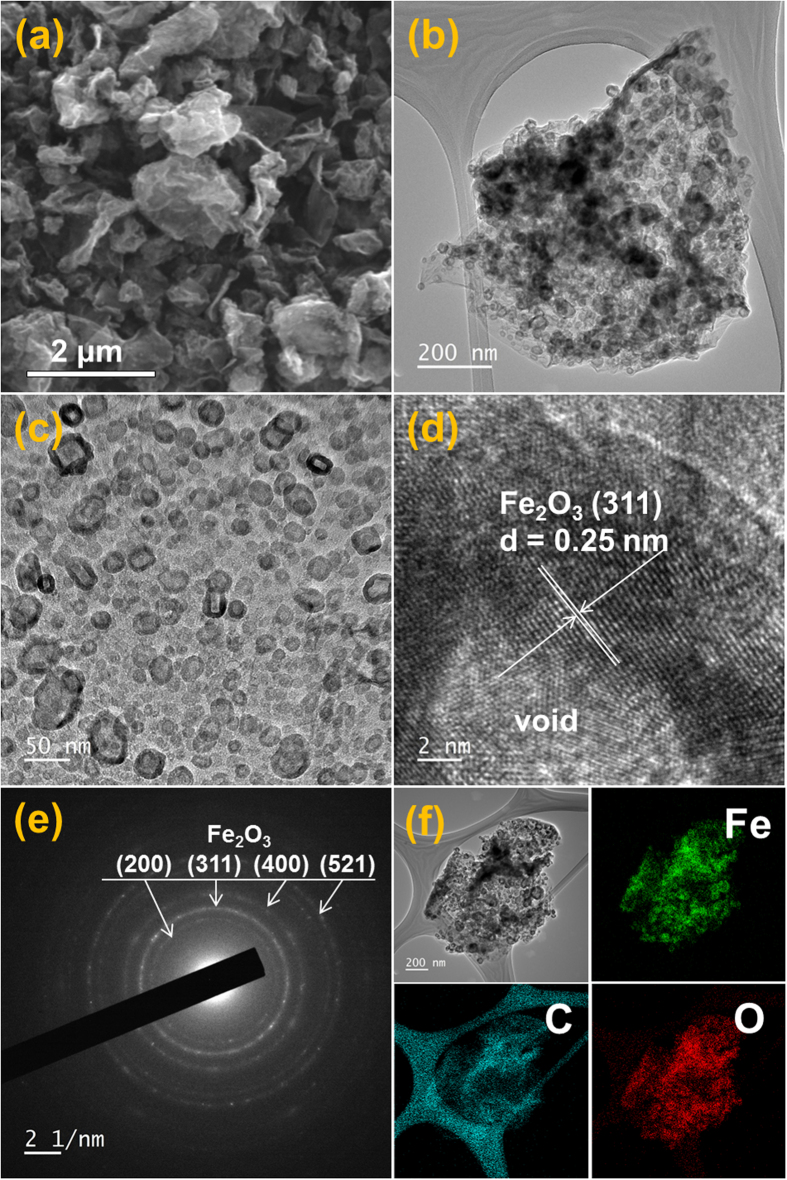
Morphologies, SAED pattern, and elemental mapping images of the hollow Fe_2_O_3_-decoreated rGO composite powders obtained by nanoscale Kirkendall diffusion: (**a**) SEM image, (**b**,**c**) TEM images, (**d**) HR-TEM image, (**e**) SAED pattern, and (**f**) elemental mapping images.

The morphologies of the Fe_2_O_3_-rGO composite powders prepared as the comparison sample are shown in Fig. 5. The metallic Fe-rGO composite was post-treated at 300 °C for 6 h in air. Metallic Fe nanocrystals transformed into hollow Fe_2_O_3_ nanoparticles by nanoscale Kirkendall diffusion. Therefore, in the TEM images shown in Fig. 5, hollow Fe_2_O_3_ nanoparticles were uniformly distributed throughout the crumpled rGO matrix. The high resolution TEM image in Fig. 5d showed clear lattice fringes separated by 0.25 nm, which correspond to the (311) crystal planes of the γ-Fe_2_O_3_ phase. The XRD pattern, SAED pattern, and elemental mapping images shown in Figs S3 and 5e,f, respectively, confirmed the formation of Fe_2_O_3_-rGO composite powders by complete oxidation.

**Figure 6:**
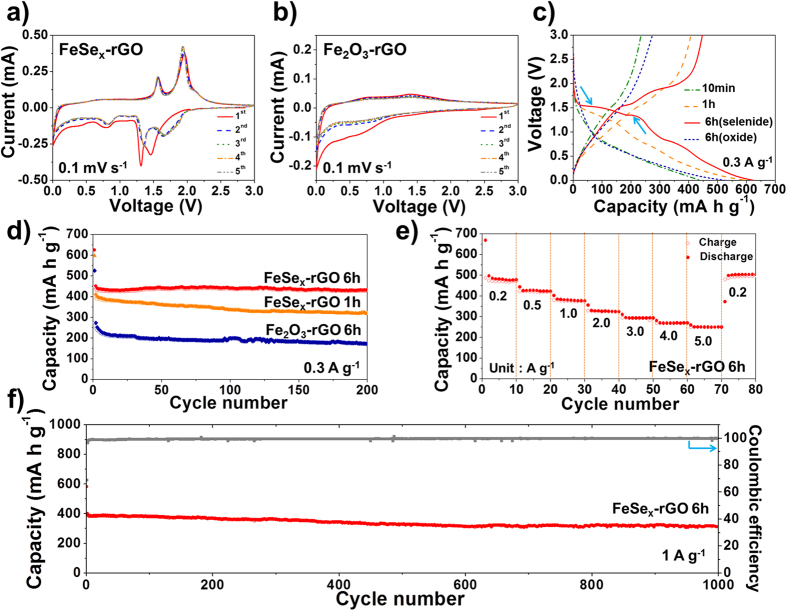
Electrochemical properties of the hollow FeSe_x_- and Fe_2_O_3_-decoreated rGO composite powders: (**a**) CV curves of FeSe_x_-rGO, (**b**) CV curves of Fe_2_O_3_-rGO, (**c**) initial charge-discharge curves, (**d**) cycling performances, (**e**) rate performance, and (**f)** long term cycling performance.

The electrochemical properties of the hollow FeSe_x_- and Fe_2_O_3_-decorated rGO composite powders for sodium-ion storage are shown in Fig. 6. The cyclic voltammograms (CVs) of the FeSe_x_-rGO and Fe_2_O_3_-rGO composite powders obtained by post-treatment in H_2_Se and air for 6 h during the first five cycles at a scan rate of 0.1 mV s^−1^ in the voltage range of 0.001–3 V are shown in Fig. 6a,b, respectively. The first cathodic scan of the FeSe_x_-rGO composite powders showed four distinct peaks located at 1.5, 1.3, 0.8, and 0.4 V. The two sharp reduction peaks located at 1.5 and 1.3 were attributed to the formation of Na_x_FeSe_2_ and Na_x_FeSe, respectively^28^. The two reduction peaks located at 0.8 and 0.4 were attributed to the formation of FeSe and Na_2_Se, and Fe and Na_2_Se, respectively^28^. During the anodic scans, two oxidation peaks were observed at 1.6 and 2.0 V, which were attributed to the formation of Na_x_FeSe_2_ and FeSe_2_, respectively^28^. The first cathodic scan of the Fe_2_O_3_-rGO composite powders shown in Fig. 6b revealed a broad weak peak at around 0.5 V, which might be attributed to the reduction of Fe (III) to Fe (0)^33^. The initial discharge and charge curves of the Fe_2_O_3_-rGO composite powders shown in Fig. 6c also did not show distinct plateaus. The initial discharge and charge curves of the composite powders obtained after selenization at different times at a constant current density of 0.3 A g^−1^ are shown in Fig. 6c. The selenization of the composite powders did not occur at a short selenization time of 10 min as confirmed by XRD pattern shown in Fig. S6. Therefore, the clear plateaus in the initial discharge and charge curves were not observed for the sample obtained after selenization for 10 min. Unclear plateaus were also observed in the initial discharge and charge curves of the FeSe_x_-rGO composite powders obtained after selenization for 1 h, in which complete selenization of the composite powders was achieved. However, the initial discharge and charge curves of the FeSe_x_-rGO composite powders obtained after selenization for 6 h showed clear plateaus, as indicated by arrows in Fig. 6c. The crystal growth of the FeSe_x_ phase during the long-term selenization process resulted in clear plateaus in their initial discharge and charge curves. The initial discharge capacities of the composite powders obtained after selenization for 10 min, 1 h, and 6 h were 444, 598, and 625 mA h g^−1^, respectively, and their corresponding charge capacities were 235, 408, and 447 mA h g^−1^, respectively. The complete conversion of the metallic Fe to the FeSe_x_ phase increased the initial discharge and charge capacities of the composite powders. The cycling performances of the composite powders obtained after selenization for different times at a constant current density of 0.3 A g^−1^ are shown in Fig. 6d. The FeSe_x_-rGO composite powders obtained after selenization for 6 h had superior cycling performance compared to that of the composite powders obtained after selenization for 1 h. The long-term selenization process for 6 h improved the sodium-ion storage properties of the FeSe_x_-rGO composite powders by improving the crystallinity. The discharge capacities of the FeSe_x_- and Fe_2_O_3_-rGO composite powders obtained after the post-treatment process for 6 h were 434 and 174 mA h g^−1^, respectively, for the 200^th^ cycle at a current density of 0.3 A g^−1^. The rate performance of the FeSe_x_-rGO composite powders obtained after selenization for 6 h is shown in Fig. 6e, in which the current density increases in a step-wise manner from 0.2 to 5 A g^−1^. The composite powders had final discharge capacities of 478, 423, 377, 324, 294, 270, and 250 mA h g^−1^ at current densities of 0.2, 0.5, 1, 2, 3, 4, and 5 A g^−1^, respectively. The discharge capacity of the composite powders recovered well to 504 mA h g^−1^ when the current density was returned to 0.2 A g^−1^ after cycling at high current densities. The long-term cycling performance of the FeSe_x_-rGO composite powders obtained after selenization for 6 h is shown in Fig. 6f for a high current density of 1 A g^−1^. The discharge capacities of the 2^nd^ and 1000^th^ cycles were 402.0 and 311.4 mA h g^−1^, respectively, and the capacity retention measured from the second cycle was 77%.

**Figure 7:**
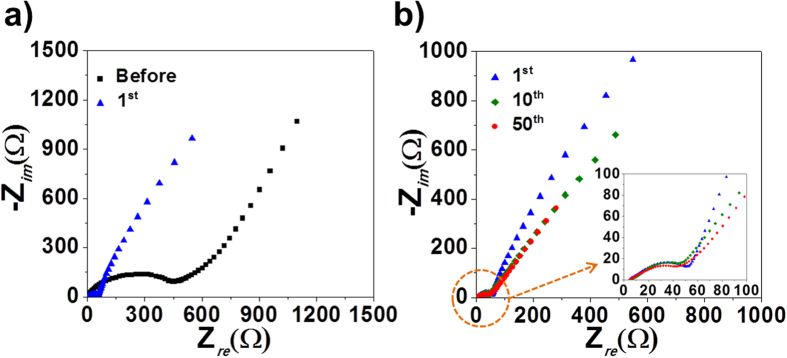
Nyquist impedance plots of the hollow FeSe_x_-decoreated rGO composite powders: (**a**) before and after 1 cycle and (**b**) after 1, 10, and 50 cycles.

The excellent sodium-ion storage properties of the FeSe_x_-rGO composite powders obtained after selenization for 6 h were confirmed by electrochemical impedance spectroscopy (EIS) measurements, as shown in Fig. 7. EIS measurements were performed on the composite powders before and after 1, 10, and 50 cycles. The Nyquist plots display compressed semicircles in the medium-frequency range, which indicate the charge-transfer resistance (*R*_*ct*_) of the electrode^34^,^35^,^36^. The charge-transfer resistance of the FeSe_x_-rGO composite powders decreased after the first cycle due to the formation of ultrafine nanocrystals during initial cycling. The low charge-transfer resistance of the electrode was maintained even after 50 cycles due to the high structural stability of the FeSe_x_-rGO composite powders for repeated sodium-ion charge and discharge processes.

## Conclusions

This paper is the first report of the synthesis of nanostructured iron selenide materials decorated within the crumpled rGO matrix and their electrochemical properties as NIB anode materials. Metallic Fe-rGO composite powders transformed into FeSe_x_-rGO composite powders by a simple selenization process with H_2_Se gas, in which the nanoscale Kirkendall diffusion process changed the densely structured Fe nanocrystals into hollow FeSe_x_ nanospheres. The FeSe_x_-rGO composite powders showed excellent long-term cycling performance at a high current density of 1 A g^−1^ and good rate performance for sodium-ion storage. The simple process developed in this study could be applied in the preparation of uniquely structured metal selenide-rGO composite powders with various compositions for wide applications including sodium-ion batteries.

## Materials and Methods

### Sample preparation

A three-step process was applied to the preparation of hollow FeSe_x_ nanoparticles-decorated rGO composite powders. Iron oxide-decorated rGO composite powders were prepared by a simple spray pyrolysis process using a spray solution of iron nitrate nonahydrate (Fe(NO_3_)_3_·9H_2_O) and graphene oxide (GO) nanosheets. GO nanosheets were synthesized from graphite flakes using a modified Hummers method, as described in our previous report^29^. As-synthesized GO nanosheets were re-dispersed in distilled water and then exfoliated by ultrasonication. 0.015 M of Fe(NO_3_)_3_·9H_2_O was dissolved in 500 mL of 1 mg mL^−1^ exfoliated GO dispersion to fabricate iron oxide-decorated rGO composite powders. In the spray pyrolysis process, droplets were generated using a 1.7-MHz ultrasonic spray generator consisting of six vibrators. The droplets were carried to a quartz reactor with length of 1200 mm and diameter of 50 mm, using N_2_ as the carrier gas at a flow rate of 10 L min^−1^. The reactor temperature was maintained at 400 °C. The first step in the post-treatment process was carried out at 400 °C for 3 h under a 10% H_2_/Ar reducing atmosphere; this produced metallic Fe nanopowder-decorated rGO composite powders. The second step in the post-treatment process of the metallic Fe nanopowder-decorated rGO composite powders was carried out at 300 °C for 6 h in H_2_Se gas, which was formed from commercial selenium metal powders by hydrogen gas, to produce hollow FeSe_x_ nanoparticles-decorated rGO composite powders. For the selenization process, the metallic Fe nanopowder-decorated rGO composite powders and selenium metal powders were loaded in a covered alumina boat and placed in a quartz tube reactor. The post-treatment process of the metallic Fe nanopowder-decorated rGO composite powders at 300 °C for 6 h under air atmosphere produced hollow Fe_2_O_3_ nanoparticles-decorated rGO composite powders.

### Characterization

The crystal structures of the composite powders were investigated using X-ray diffractometry (XRD, X’pert PRO MPD) with Cu-K_α_ radiation (λ = 1.5418 Å) at the Korea Basic Science Institute (Daegu). The morphologies of the powders were investigated using field-emission scanning electron microscopy (FE-SEM, Hitachi S-4800) and high-resolution transmission electron microscopy (HR-TEM, JEOL JEM-2100F) at a working voltage of 200 kV. X-ray photoelectron spectroscopy (XPS) of the powders was performed using an ESCALAB-250 with Al K_α_ radiation (1486.6 eV). To determine the amount of rGO in the hollow FeSe_x_ nanoparticles-decorated rGO composite powders, thermogravimetric analysis (TGA, SDT Q600) was performed in air at a heating rate of 10 °C min^−1^.

### Electrochemical measurements

The electrochemical properties of the powders were analyzed using a 2032-type coin cell. The anode was prepared by mixing the active material, carbon black, and sodium carboxymethyl cellulose in a weight ratio of 7:2:1. Stick-type sodium metal and microporous polypropylene film were used as the counter electrode and separator, respectively. The separators (Welcos Co., Ltd., Korea) were microporous monolayer polypropylene membranes, with a thickness of 22 μm. The electrolyte was 1 M NaClO_4_ (Aldrich) dissolved in a mixture of ethylene carbonate/dimethyl carbonate (EC/DMC; 1:1 v/v, PURIEL), to which 5 wt% fluoroethylene carbonate (FEC) was added. The discharge/charge characteristics of the samples were investigated by cycling in the potential range of 0.001–3 V at various current densities. Cyclic voltammograms (CVs) were measured at a scan rate of 0.1 mV s^−1^. The dimensions of the anode were 1 cm × 1 cm and the mass loading was approximately 1.2 mg cm^−2^. The electrochemical impedance was measured using electrochemical impedance spectroscopy (EIS) over the frequency range of 0.01 Hz–100 kHz.

## Additional Information

**How to cite this article**: Park, G. D. *et al*. Na-ion Storage Performances of FeSe_x_ and Fe_2_O_3_ Hollow Nanoparticles-Decorated Reduced Graphene Oxide Balls prepared by Nanoscale Kirkendall Diffusion Process. *Sci. Rep.* **6**, 22432; doi: 10.1038/srep22432 (2016).

## Supplementary Material

Supplementary Information
