# Supplementary material for: Na-ion Storage Performances of FeSex and Fe2O3 Hollow Nanoparticles-Decorated Reduced Graphene Oxide Balls prepared by Nanoscale Kirkendall Diffusion Process
Source: Sci Rep. 2016 Feb 29;6:22432. doi: 10.1038/srep22432 (PMC4774857; doi:10.1038/srep22432)
Supplement: Supplementary Information [file srep22432-s1.pdf]

Supporting Information

Na-ion Storage Performances of $FeSe_x$ and Fe_2O_3 Hollow Nanoparticles-Decorated Reduced Graphene Oxide Ball prepared by Nanoscale Kirkendall Diffusion process

Gi Dae Park[1], Jung Sang Cho[1], Jung-Kul Lee[2,], and Yun Chan Kang[1,]**

[1]Department of Materials Science and Engineering, Korea University, Anam Dong, Seongbuk-Gu, Seoul 136-713, Republic of Korea

[2]Department of Chemical Engineering, Konkuk University, 1 Hwayang-Dong, Gwangjin-Gu, Seoul 143-701, Republic of Korea

*Corresponding authors. E-mail: yckang@korea.ac.kr (Y. C. Kang), jkrhee@konkuk.ac.kr (J.-K. Lee)

Keywords: Kirkendall diffusion, hollow nanoparticles, metal selenide, sodium ion batteries, spray pyrolysis

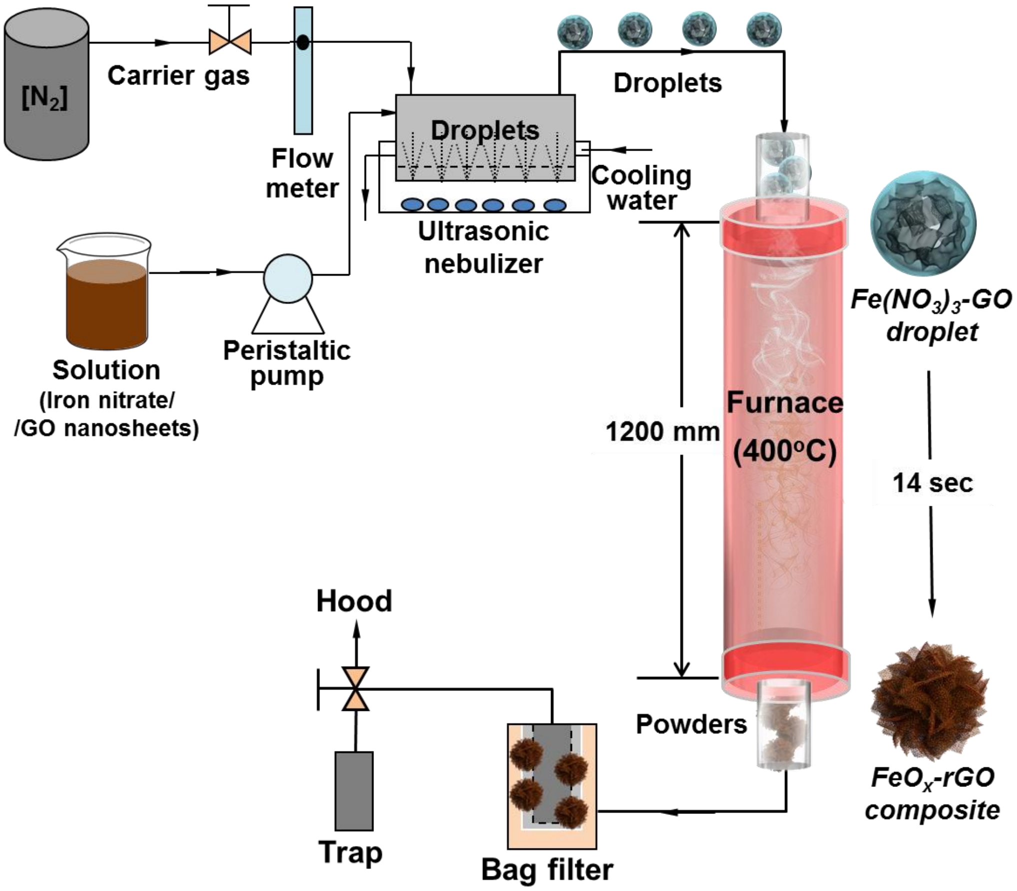

Figure S1 Schematic diagram of the spray pyrolysis system for the FeO_x-rGO composite powders.

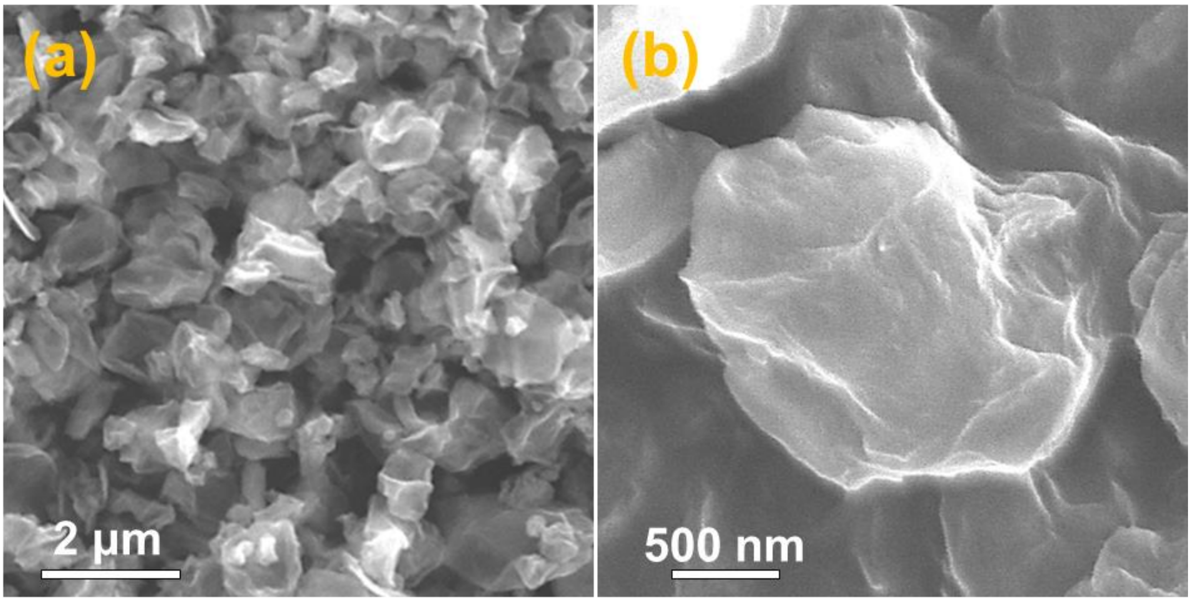

Figure S2 Morphologies of the iron oxide-decorated rGO composite powders directly prepared by spray pyrolysis: (a) low magnification and (b) high magnification SEM images.

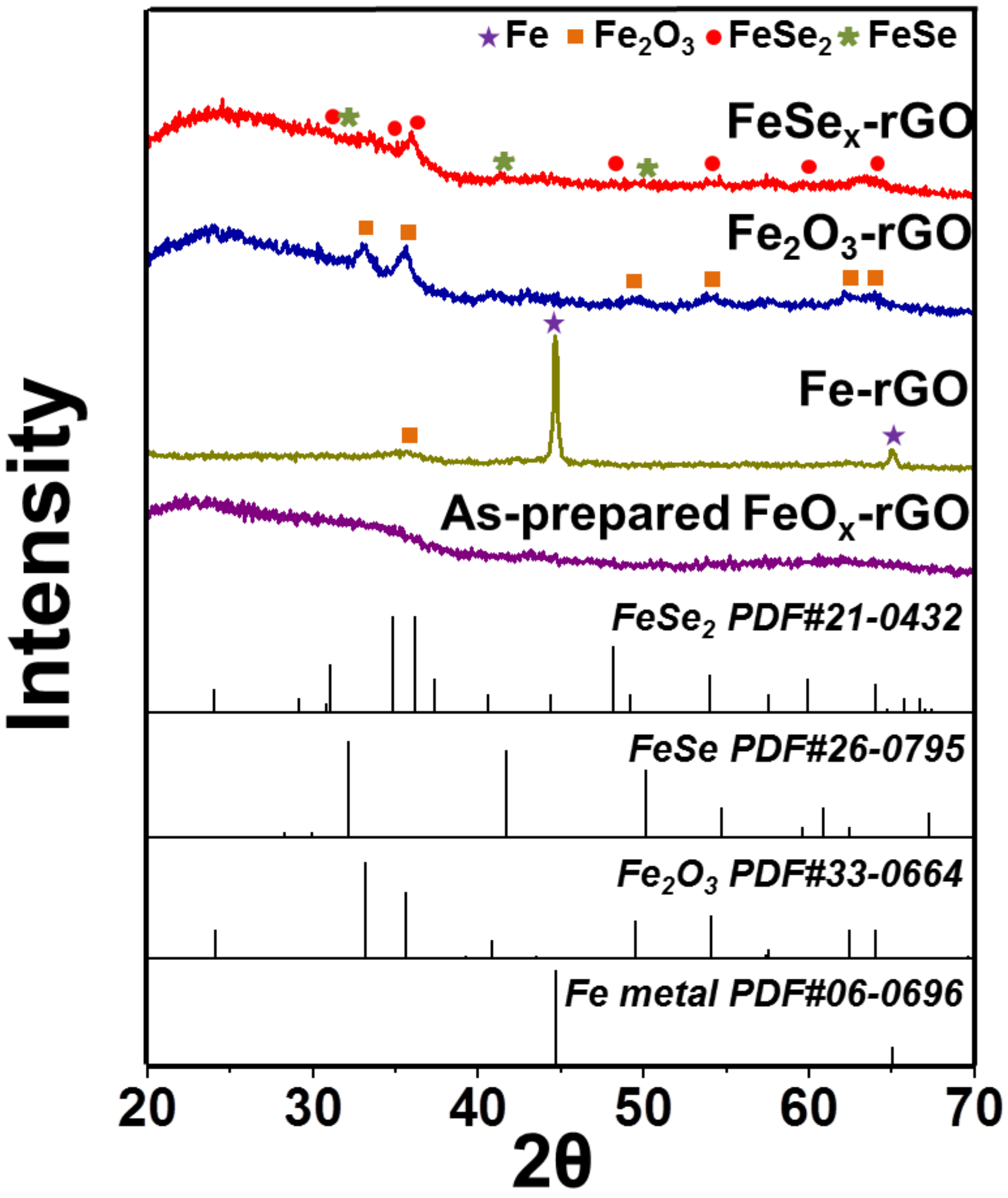

Figure S3 XRD patterns of the Fe-, $FeSe_x$- and Fe_2O_3-decorated rGO composite powders.

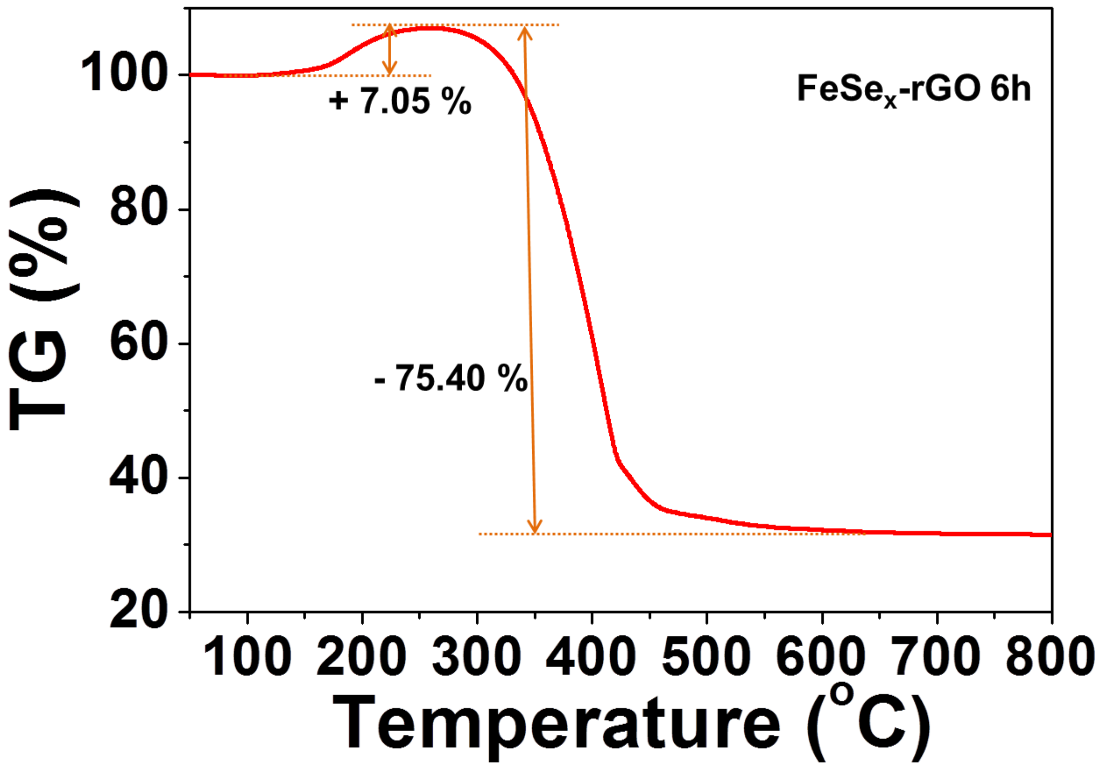

Figure S4 TG curve of the $FeSe_x$-decorated rGO composite powders.

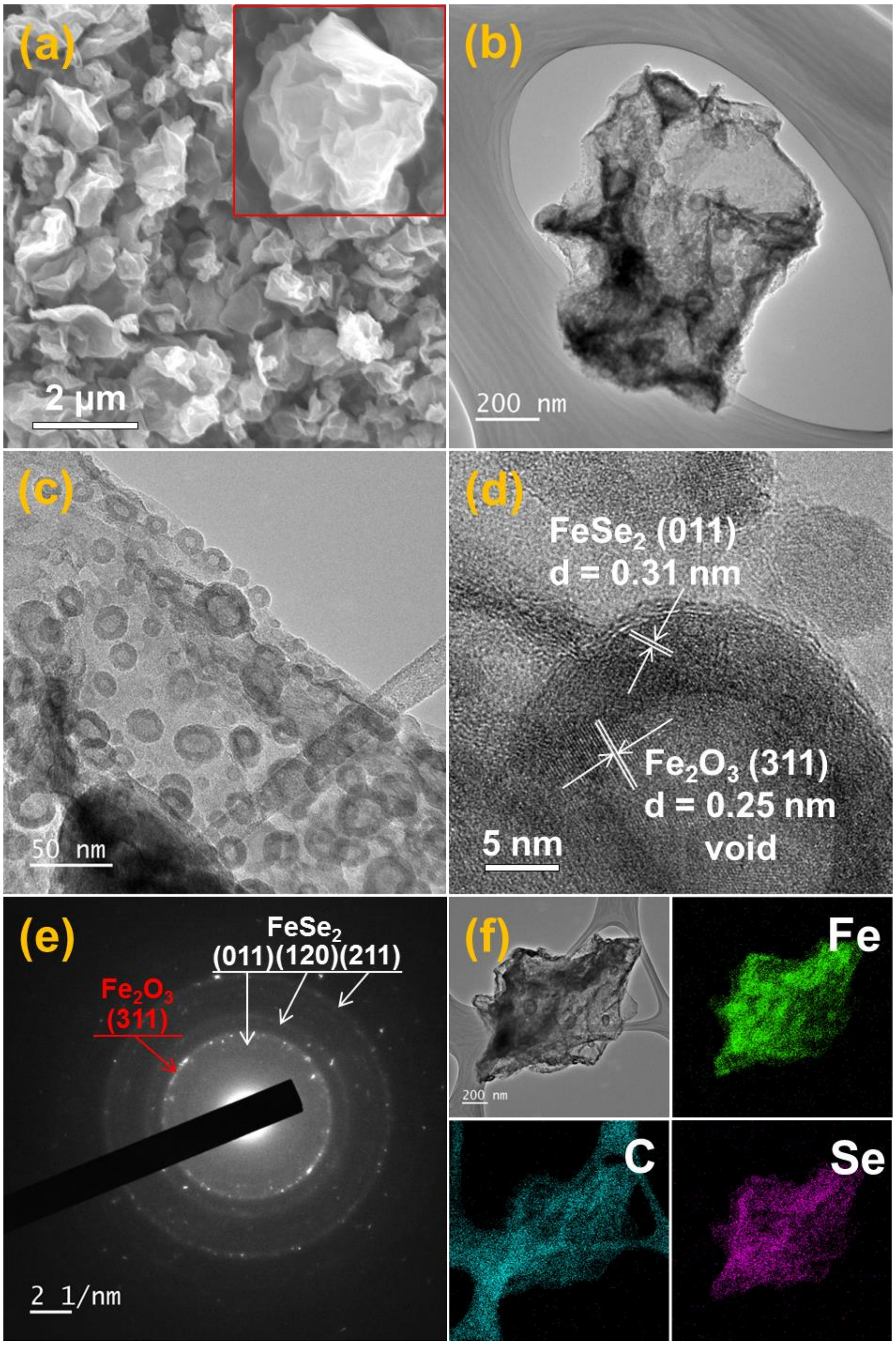

Figure S5 Morphologies, SAED pattern, and elemental mapping images of the composite powder obtained after short time selenization process of 1 h: (a) SEM image, (b), (c) TEM images, (d) HR-TEM image, (e) SAED pattern, and (f) elemental mapping images.

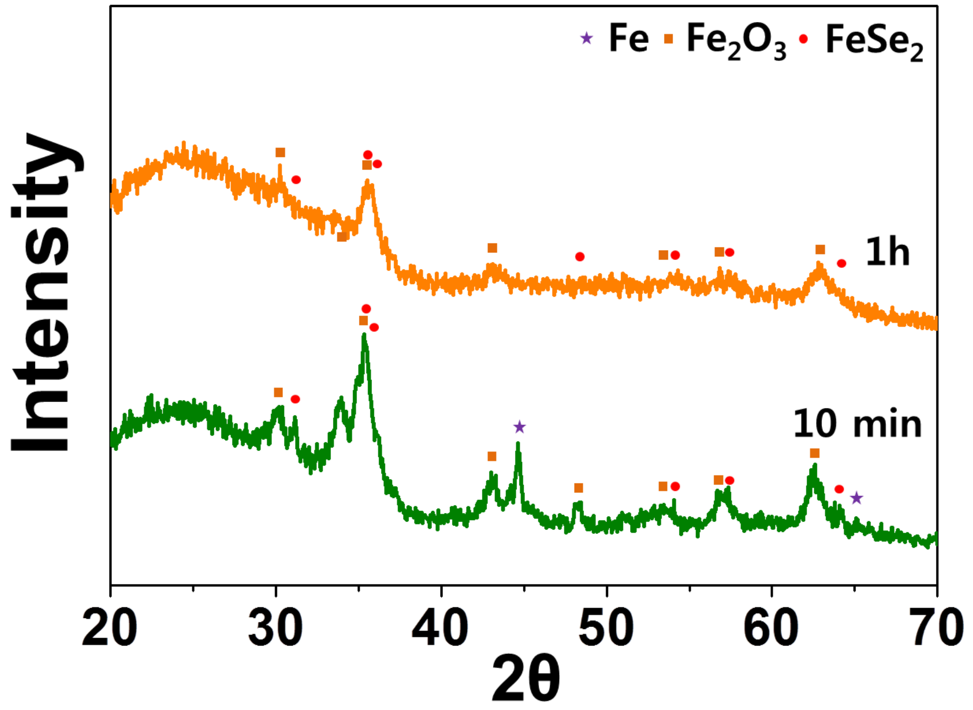

Figure S6 XRD patterns of the composite powders obtained after selenization for 10 min and 1 h.
